# Supplementary material for: Pathogen effector recognition-dependent association of NRG1 with EDS1 and SAG101 in TNL receptor immunity
Source: Nat Commun. 2021 Jun 7;12:3335. doi: 10.1038/s41467-021-23614-x (PMC8185089; doi:10.1038/s41467-021-23614-x)
Supplement: Supplementary file 1 — Supplementary Information [file 41467_2021_23614_MOESM1_ESM.pdf]

SUPPLEMENTARY INFORMATION

For the study Sun, Lapin, Feehan et al. (2021)

“Pathogen effector recognition-dependent association of NRG1 with EDS1 and SAG101 in TNL receptor immunity”

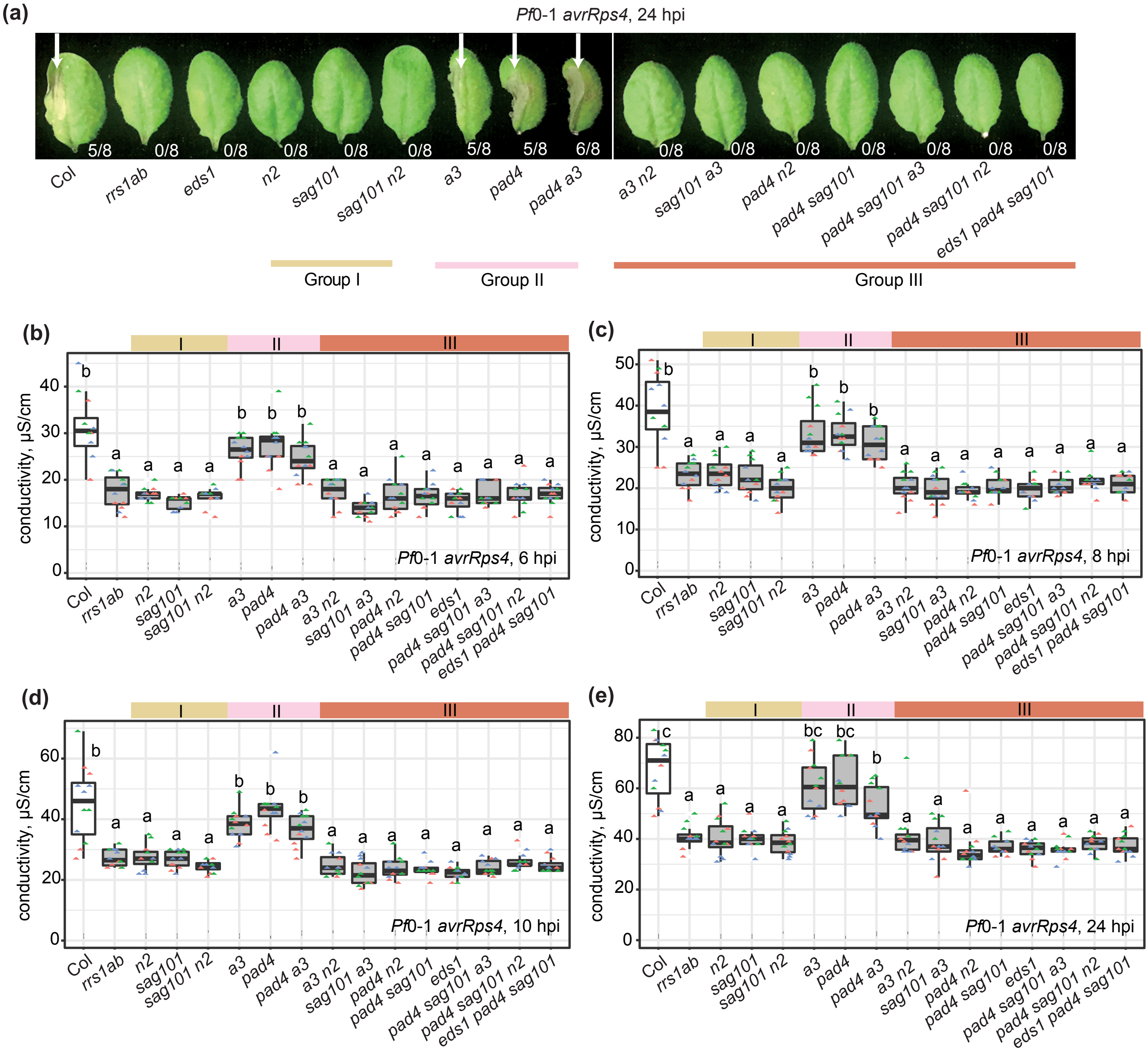

Supplementary Figure 1. No evidence for the cross-use of components between the *PAD4-ADR1* and *SAG101-NRG1* branches in *Arabidopsis* TNL$^{RRS1-RPS4}$ cell death. Related to Fig. 1 **(a)** Macroscopic symptoms of TNL$^{RRS1-RPS4}$ cell death triggered by *Pf*0-1 *avrRps4* in Col-0 (Col), *rrs1a rrs1b* (*rrs1ab*), and genotypes with mutated *SAG101* and/or *NRG1*s (group I), *PAD4* and/or *ADR1*s (group II) or their cross-branch combinations (group III). White arrow indicates cell death visible as tissue collapse at 24 hours post bacteria infiltration (hpi). Numbers indicate number of leaves with visible tissue collapse from the total number of infiltrated leaves from four plants in one experiment. The experiment was conducted three times with similar results. **(b-e)** A boxplot presentation of electrolyte leakage data quantifying cell death at 6 **(b)**, 8 **(c)**, 10 **(d)** and 24 **(e)** hpi with *Pf*0-1 *avrRps4* (OD_{600}=0.2) for indicated genotypes. Inference of statistically significant differences was based on Tukey's HSD test (α=0.001, n=12). Description of boxplots: minima - first quartile, maxima - third quartile, center – median, whiskers extend to minimum and maximum values but not further than 1.5 inter-quartile range from the respective minima or maxima of the boxplot. Datapoints with the same colour are from one experiment. *PAD4* and *SAG101* do not form signalling branches with *NRG1*s and *ADR1*s, respectively, to promote receptor TNL-dependent (RRS1-RPS4) cell death. Abbreviations: *Pf*0-1 – effector tester strain of *Pseudomonas fluorescens* 0-1, hpi – hours post inoculation. Grouping of genotypes: mutants with mutated *SAG101* and/or *NRG1*s (group I), *PAD4* and/or *ADR1*s (group II) or their cross-branch combinations (group III).

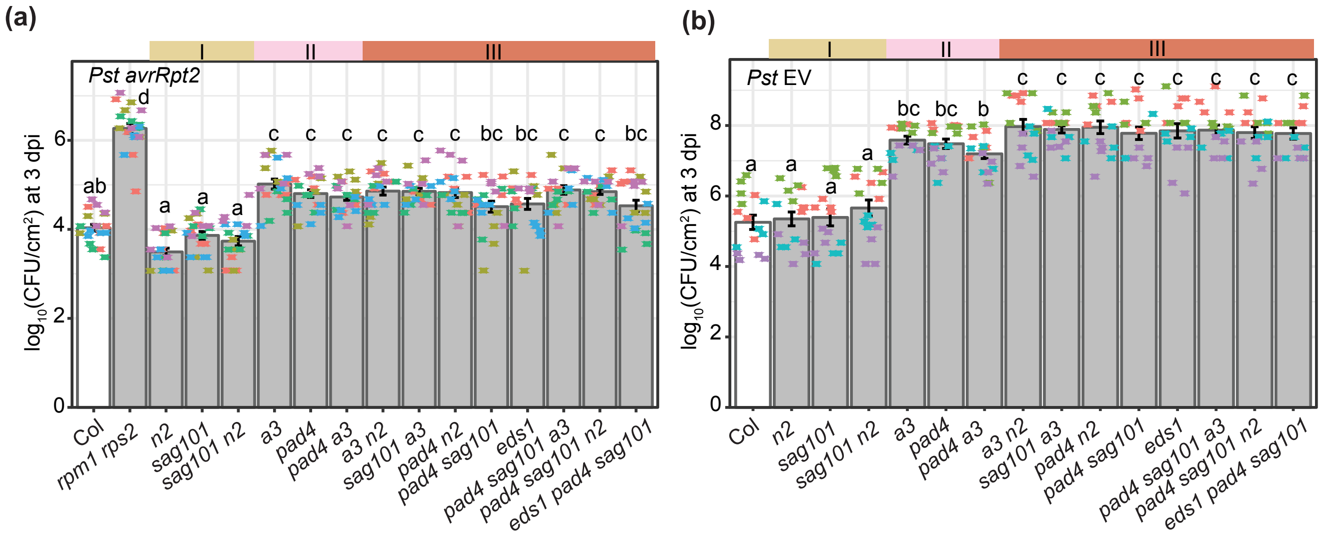

Supplementary Figure 2. No evidence for the cross-use of components between the *PAD4-ADR1* and *SAG101-NRG1* branches in *Arabidopsis* basal and CNL^{RPS2} bacterial resistance. Related to Fig. 1. Jitter and barplot presentation of *Pst avrRpt2* **(a)** and *Pst* empty vector (EV) **(b)** growth in indicated genotypes within groups I, II and III at 3 days after inoculation (dpi) via syringe infiltration (OD_{600}=0.0005). Bacterial loads are shown as log_{10} colony-forming units (CFU) per cm^2. Experiments were performed three times independently with four replicates each (Tukey's HSD, α =0.001, n=12). Error bars represent standard error of mean. Datapoints with the same colour are from one experiment. *PAD4* and *SAG101* do not form signalling branches with *NRG1*s and *ADR1*s, respectively, to promote receptor TNL-dependent (RRS1-RPS4) cell death. Abbreviations: *Pst* - *Pseudomonas syringae* pv. *tomato* DC3000. Grouping of genotypes: mutants with mutated *SAG101* and/or *NRG1*s (group I), *PAD4* and/or *ADR1*s (group II) or their cross-branch combinations (group III).

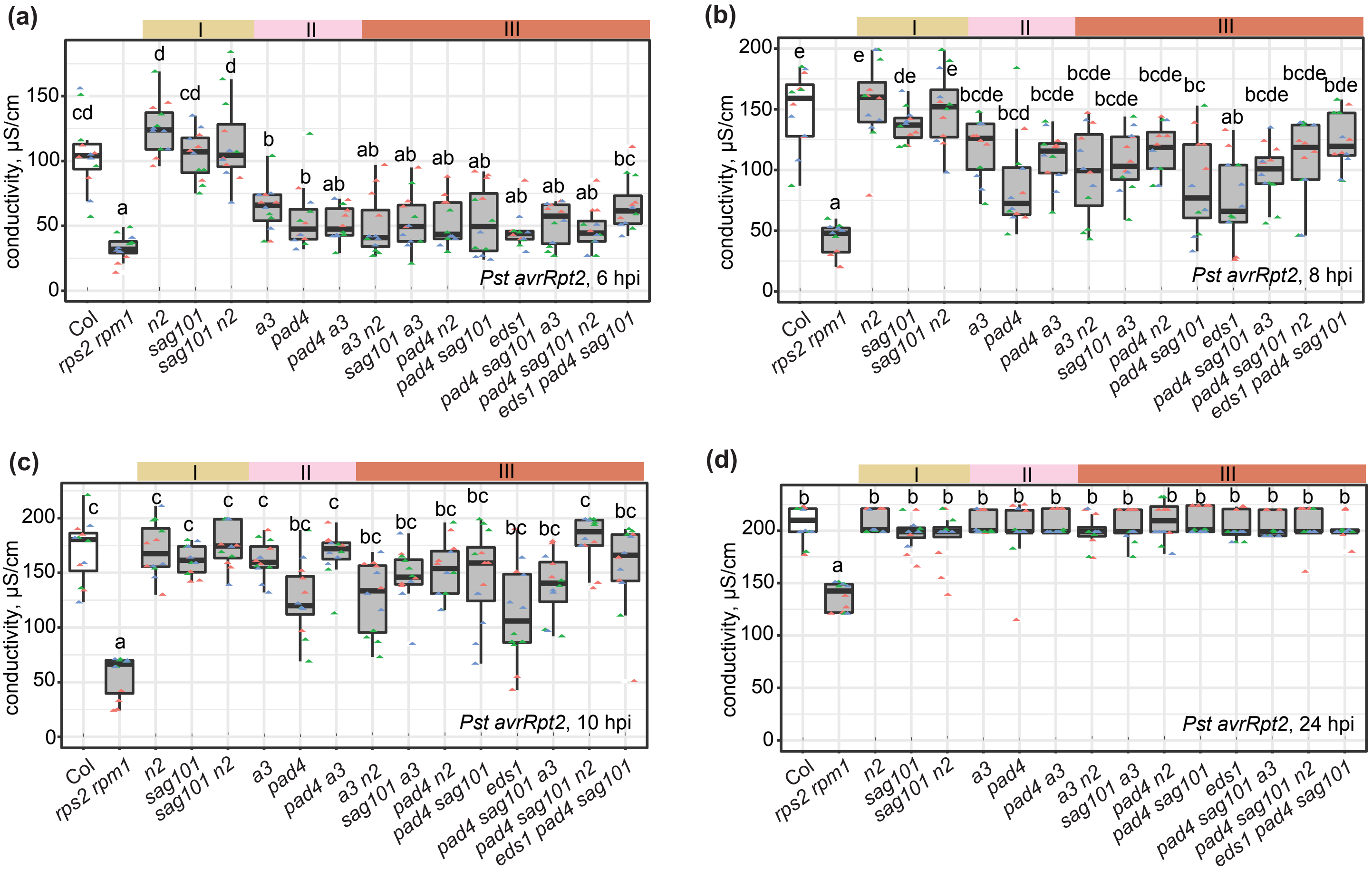

Supplementary Figure 3. No evidence for the cross-use of components between the *PAD4-ADR1* and *SAG101-NRG1* branches in *Arabidopsis* CNL^{RPS2} cell death. Related to Fig. 1. A boxplot presentation of electrolyte leakage data quantifying cell death at 6 **(a)**, 8 **(b)**, 10 **(c)** and 24 **(d)** hpi with *Pst avrRpt2* (OD_{600}=0.02) for indicated genotypes within groups I, II and III. Statistical significance of difference was assessed with Tukey's HSD test (α=0.001, n=12, three independent experiments). Description of boxplots: minima - first quartile, maxima - third quartile, center – median, whiskers extend to minimum and maximum values but not further than 1.5 inter-quartile range from the respective minima or maxima of the boxplot. Datapoints with the same colour are from one experiment. *PAD4* and *SAG101* do not form signalling branches with *NRG1*s and *ADR1*s, respectively, to promote receptor CNL-dependent (RPS2) cell death. Abbreviations: *Pst* - *Pseudomonas syringae* pv. *tomato* DC3000, hpi – hours post inoculation. Grouping of genotypes: mutants with mutated *SAG101* and/or *NRG1*s (group I), *PAD4* and/or *ADR1*s (group II) or their cross-branch combinations (group III).

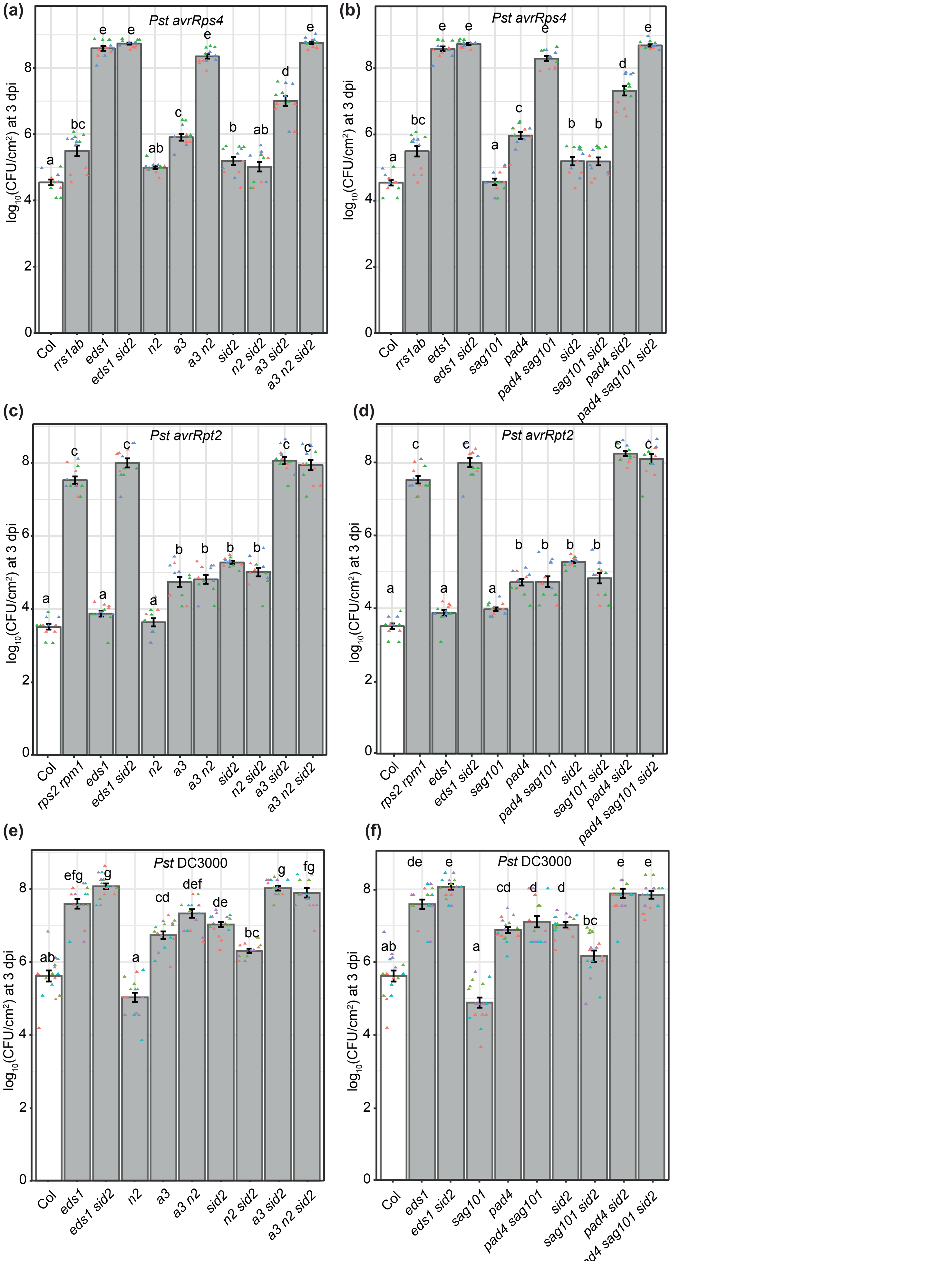

Supplementary Figure 4. Growth of *Pst avrRps4* (a, b), *Pst avrRpt2* (c, d) and *Pst* (empty vector, (e, f)) in mutants defective in *ICS1/SID2* and *PAD4-ADR1*s or *SAG101-NRG1*s branches. Related to Fig. 2a. Bacterial loads are expressed as colony forming units (CFU) per cm^2 at 3 dpi on a log_{10} scale. Bacteria were syringe-infiltrated (OD_{600}=0.0005). The experiments were performed three times with four technical replicates (leaf discs) each (Tukey's HSD, α=0.001, n=12). Error bars represent standard error of mean. Datapoints with the same colour were recorded in one experiment.

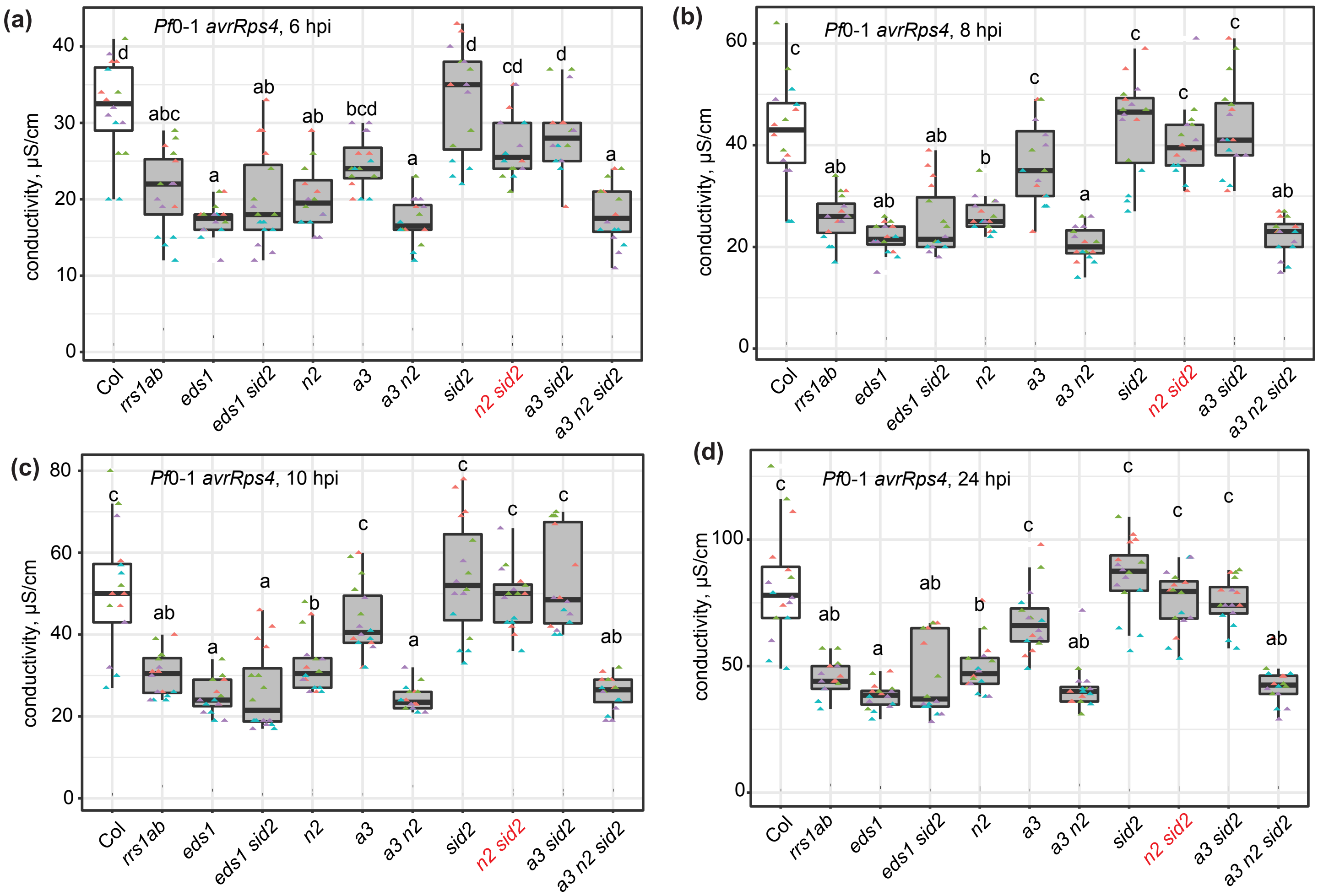

Supplementary Figure 5. Removal of *NRG1*s and *ICS1* reveals an *ADR1*-dependent *Arabidopsis* TNL$^{RRS1\text{-}RPS4}$ early cell death branch. Related to Fig. 2b. Boxplot presentation of *Pf*0-1 *avrRps4*-triggered electrolyte leakage for indicated genotypes at 6 **(a)**, 8 **(b)**, 10 **(c)**, and 24 **(d)** hpi. Genotypes shown in Supplementary Figures 5 and 6 were tested simultaneously but are split to enhance visualization. Significance codes were assigned for genotypes shown in the panels based on Tukey's HSD test (α=0.001, n=16, four independent experiments). Description of boxplots: minima - first quartile, maxima - third quartile, center – median, whiskers extend to minimum and maximum values but not further than 1.5 inter-quartile range from the respective minima or maxima of the boxplot. Data for control lines (Col-0, *rrs1a rrs1b*, *eds1*, *eds1 sid2*) were reused to calculate statistics in left and right panels. Genotypes with mutated *NRG1.1* and *NRG1.2* (*n2*) in red display cell death after *Pf*0-1 *avrRps4* infiltration. Abbreviations: *Pf*0-1 – effector tester strain of *Pseudomonas fluorescens* 0-1, hpi – hours post inoculation. Datapoints with the same colour were recorded in one experiment.

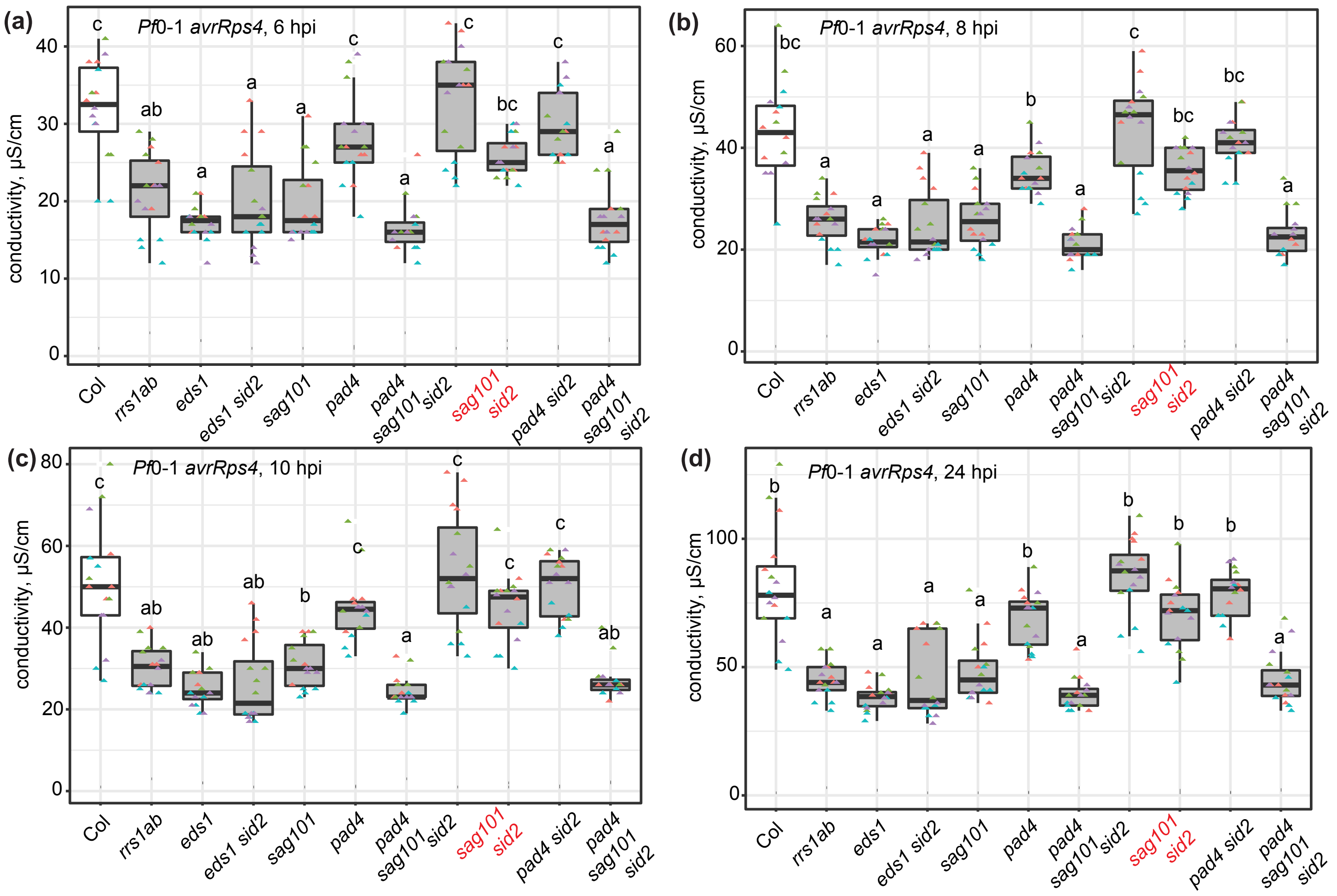

Supplementary Figure 6. Removal of *SAG101* and *ICS1* reveals a *PAD4*-dependent *Arabidopsis* TNL$^{RRS1\text{-}RPS4}$ early cell death branch. Related to Fig. 2b. Boxplot presentation of *Pf*0-1 *avrRps4*-triggered electrolyte leakage for indicated genotypes at 6 **(a)**, 8 **(b)**, 10 **(c)**, and 24 **(d)** hpi. Genotypes shown in Supplementary Figures 5 and 6 were tested simultaneously but are split to enhance visualization. Significance codes were assigned for genotypes shown in the panels based on Tukey's HSD test (α=0.001, n=16 from four independent experiments). Description of boxplots: minima - first quartile, maxima - third quartile, center – median, whiskers extend to minimum and maximum values but not further than 1.5 inter-quartile range from the respective minima or maxima of the boxplot. Data for control lines (Col-0, *rrs1a rrs1b*, *eds1*, *eds1 sid2*) were reused to calculate statistics in left and right panels. Genotypes with mutated *SAG101* in red display cell death after *Pf*0-1 *avrRps4*. Abbreviations: *Pf*0-1 – effector tester strain of *Pseudomonas fluorescens* 0-1, hpi – hours post inoculation. Datapoints with the same colour are from one independent experiment.

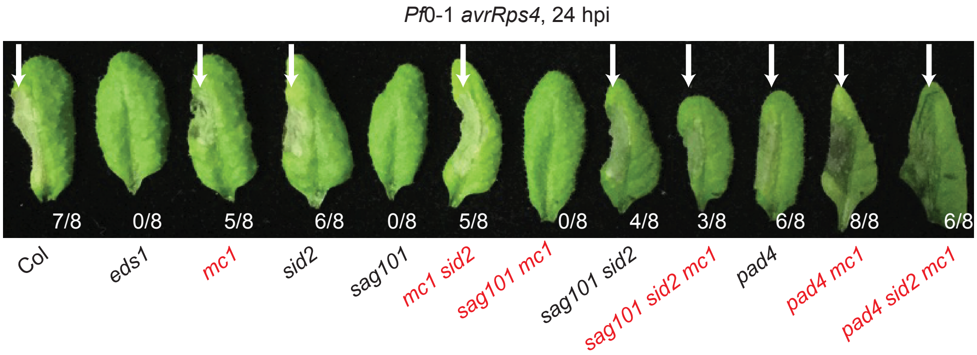

Supplementary Figure 7. *Metacaspase 1* (*MC1*) is dispensable for the TNL$^{RRS1\text{-}RPS4}$ cell death involving *SAG101-NRG1*s or *PAD4-ADR1*s. Macroscopic symptoms of cell death triggered by *Pf*0-1 *avrRps4* (OD_{600}=0.2) in Col-0 (Col) single (*eds1*, *sag101*, *pad4*, *sid2*, *mc1*) and indicated combinatorial mutants. White arrows point to collapsed leaf areas at 24 hpi. Genotypes in red contain the *mc1* mutation. Eight leaves from four plants were syringe-infiltrated with *Pf*0-1 *avrRps4*, and the number of collapsed leaves was counted at 24 hpi. The experiment was repeated three times with similar results. Abbreviations: *Pf*0-1 – effector tester strain of *Pseudomonas fluorescens* 0-1, hpi – hours post inoculation.

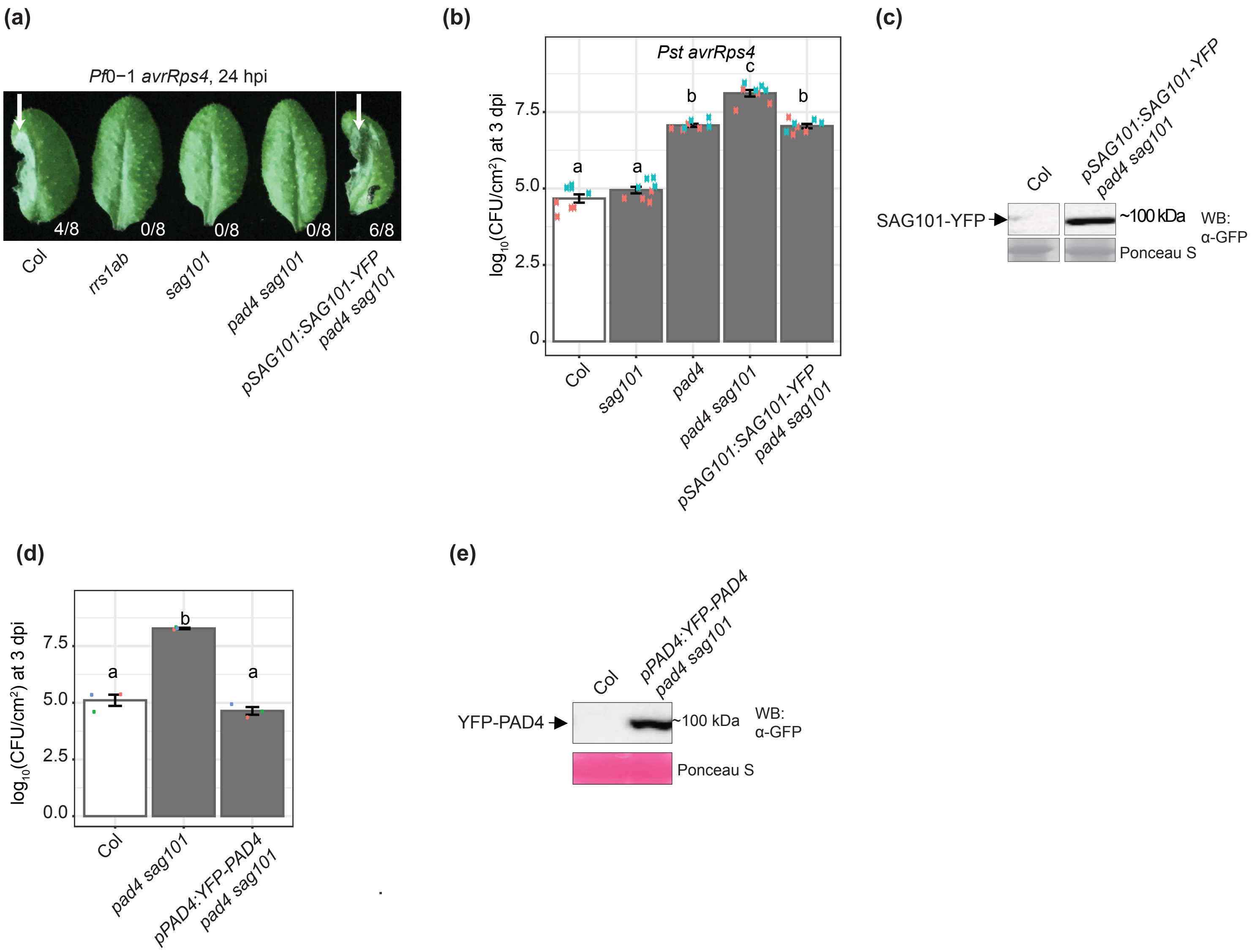

Supplementary Figure 8. Characterization of the complementation lines carrying *pSAG101:SAG101-YFP* and *pPAD4:YFP-PAD4* in the Col-0 *pad4 sag101* background. (a, b) Complementation in the *pSAG101:SAG101-YFP* (*pad4 sag101*) T3 homozygous line was assessed using RRS1-RPS4 cell death (a) and bacterial resistance (b) assays with Col-0 (Col), *rrs1a rrs1b* (*rrs1ab*), *sag101* and *pad4 sag101* as controls. **(a)** Cell death was examined visually at 24 hpi of *Pf*0-1 *avrRps4*. Numbers refer to the number of leaves showing tissue collapse vs. all infiltrated leaves. The experiment was conducted three times with similar results. **(b)** *Pst avrRps4* titers (log_{10} of CFU/cm^2) were determined at 3 dpi after bacteria syringe-infiltration (OD_{600}=0.0005). The experiment was performed twice with four replicates each (Tukey's HSD, α=0.001, n=8). Error bars represent standard error of mean. Datapoints with the same colour come from one independent experiment. Both SAG101-dependent RPS4-RRS1 cell death and resistance were recovered by the *pSAG101:SAG101-YFP* construct transformed into the signalling-defective *pad4 sag101* mutant. **(c)** Western blot analysis SAG101-YFP steady-state levels in the transgenic line in (a, b) using α-GFP antibodies. Bands were cropped from same blot. The experiment was performed twice. The SAG101-YFP fusion protein produced a band of the indicated expected size. **(d)** *Pst avrRps4* titers in *pPAD4:YFP-PAD4 pad4 sag101* T3 homozygous line at 3 dpi (OD_{600}=0.0005; syringe infiltration) (three independent experiments, one replicate from each, Tukey's HSD, α=0.001, n=3). Error bars represent standard error of mean. (e) Immunoblot analysis of PAD4-YFP steady-state levels in the transgenic line in (d) using α-GFP antibody. The detection was conducted two times with similar results. Abbreviations: *Pst* - *Pseudomonas syringae* pv. *tomato* DC3000, *Pf*0-1 – effector tester strain of *Pseudomonas fluorescens* 0-1, hpi – hours post inoculation, CFU – colony-forming units, WB – Western blotting.

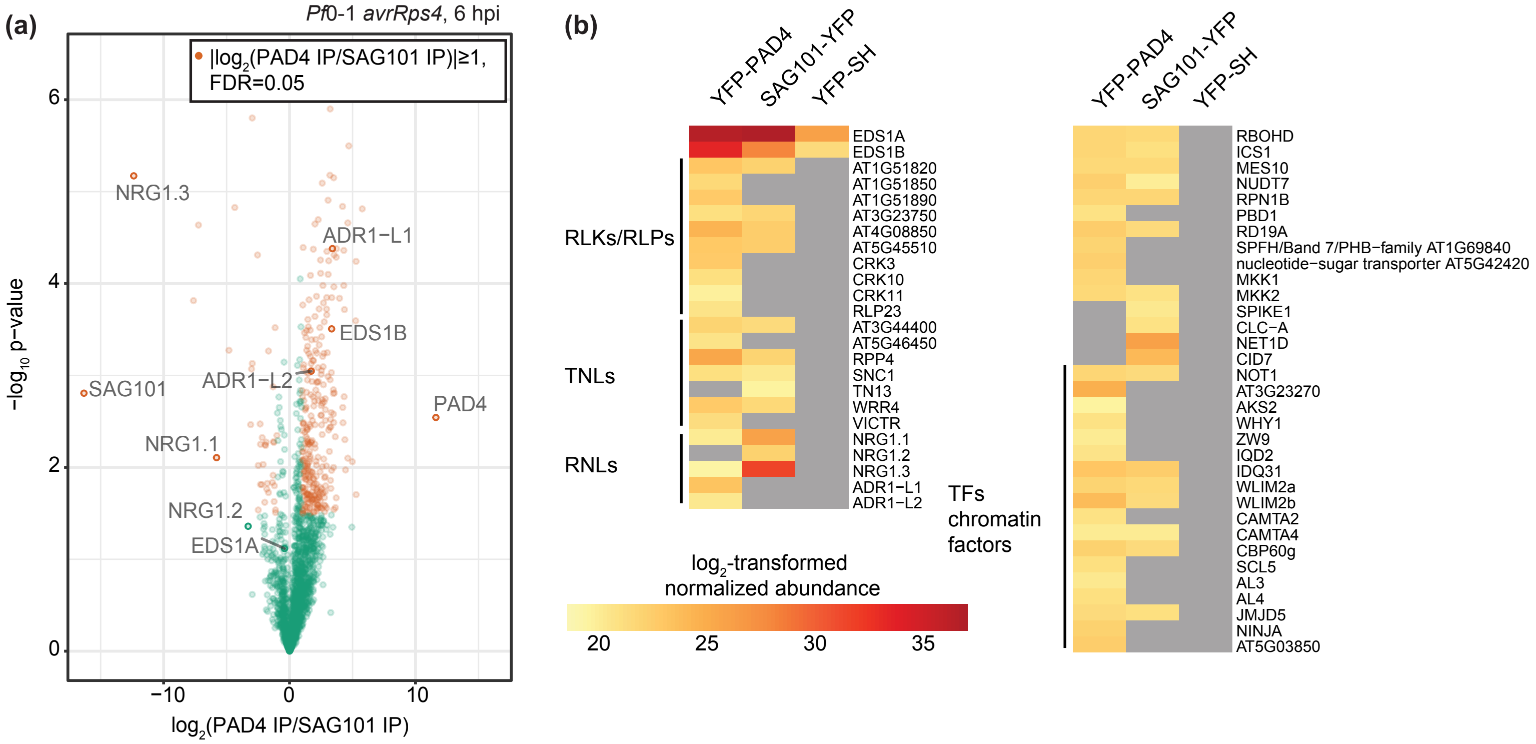

Supplementary Figure 9. Selective enrichment of RNLs with SAG101 and PAD4 in *Arabidopsis* leaves upon activation of TNL$^{RRS1\text{-}RPS4}$. (a) Volcano plot of normalized abundances (label free quantification (LFQ), log_2 scale) for proteins copurified with YFP-PAD4 and SAG101-YFP from total leaf extracts of the respective complementation lines *pPAD4:YFP-PAD4* and *pSAG101:SAG101-YFP* (both Col-0 *pad4 sag101* background) infiltrated with *Pf*0-1 *avrRps4* (6 hpi, OD_{600}=0.2). Proteins enriched in PAD4-YFP vs. SAG101-YFP IPs are shown in orange (($|log_2$(PAD4 IP/SAG101 IP)$| \geq 1$, permutation-based FDR = 0.05). Missing values were imputed. NRG1.1, NRG1.2 and NRG1.3 are enriched in the SAG101-YFP samples, while ADR1 and ADR1-L1 were detected only on PAD4-YFP samples. NRG1.2 is specifically enriched with SAG101-YFP (three of four replicates, not detected in YFP-SH and YFP-PAD4 IP samples), but it was not marked as enriched in the output of statistical analysis due to imputation. The IP-MS analysis was performed on samples collected in four independent experiments. **(b)** A heatmap of normalised estimated abundances of selected proteins specifically copurified with YFP-PAD4 and SAG101-YFP (detected in three of four replicates) but not YFP-StrepII-3xHA (YFP-SH, detected in up to one replicate) in *Arabidopsis* plants infiltrated with *Pf*0-1 *avrRps4* (6 hpi). The undetected proteins are shown in grey. YFP-tagged PAD4 and SAG101 specifically copurified several receptor-like proteins and kinases, TNLs, chromatin regulatory and transcription factors, as well as several enzymes such as NUDT7. Abbreviations: *Pf*0-1 – effector tester strain of *Pseudomonas fluorescens* 0-1, hpi – hours post inoculation, IP - immunoprecipitation.

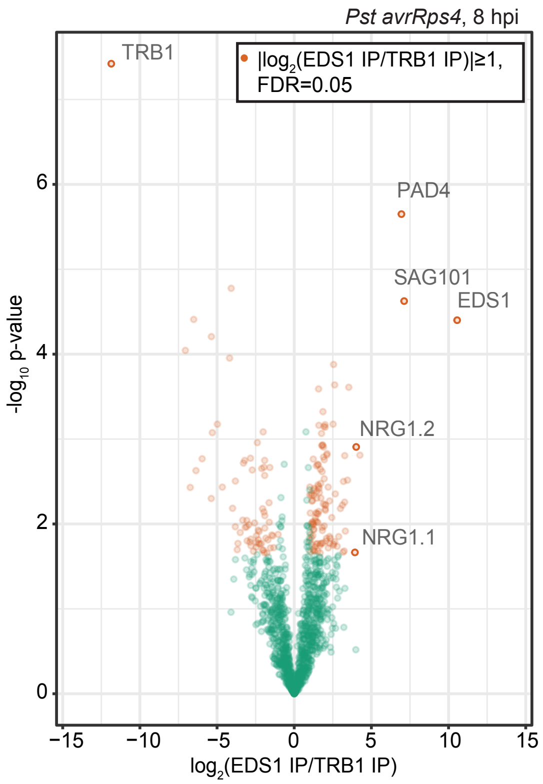

Supplementary Figure 10. NRG1 proteins specifically co-purify with EDS1 in *Arabidopsis* leaves upon activation of TNL-$^{\text{RRS1-RPS4}}$. Volcano plot of normalized abundances (LFQ, $\log_2$ scale) of proteins detected in mass-spectrometry (MS) analyses after immunoprecipitation (IP) of EDS1-YFP and TRB1-GFP from nuclear extracts of corresponding *Arabidopsis* complementation lines infiltrated with *Pst avrRps4* (8 hpi, OD_{600}=0.1). In orange are proteins enriched in EDS1-YFP vs. TRB1-GFP samples ($|\log_2$(EDS1-YFP/TRB1-GFP)$| \geq 1$, permutation-based FDR=0.05; missing values imputed). Samples for the MS analysis were collected in four independent experiments. NRG1.1 and NRG1.2 are specifically enriched in EDS1-YFP samples. Abbreviations: *Pst* - *Pseudomonas syringae* pv. *tomato* DC3000, hpi – hours post inoculation, IP - immunoprecipitation.

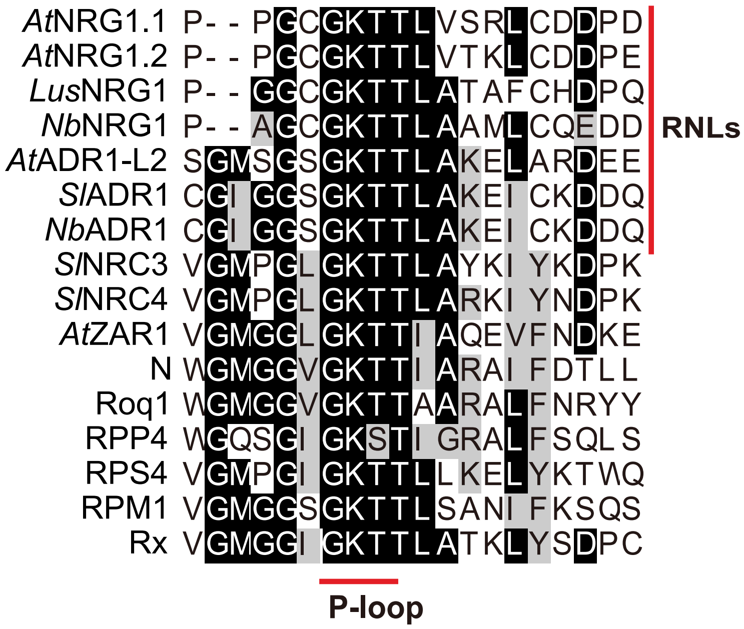

Supplementary Figure 11. Multiple sequence alignments of selected helper RPW8 domain NLRs (RNLs), helper CNLs (NRC), receptor CNLs and TNLs over the P-loop motif sequences. The P-loop is indicated with red line. Multiple sequence alignments were built (Clustal Omega) and visualised with the msa package in R. Accession numbers of NLRs in the alignment: NRG1.1 – AT5G66900.1, NRG1.2 – AT5G66910.1, *Lus*NRG1 - Lus10022464, *Nb*NRG1 - Niben101Scf02118g00018.1, *At*ADR1-L2 - AT5G04720.1, *Sl*ADR1 - Solyc04g079420.3.1, *Nb*ADR1 - Niben101Scf02422g02015.1, *Sl*NRC4 - Solyc04g007070.3.1, *Sl*NRC3 - XP_004238948.1, *At*ZAR1 - AT3G50950.2, N - Q40392, Roq1 - ATD14363.1, RPP4 - F4JNA9, RPS4 - Q9XGM3, RPM1 - Q39214, Rx - Q9XGF5.

Supplementary Table 1. Plant genetic materials used in this study

Denoted name	Full name	Description, reference
eds1	Col-0/L*er eds1-2* (multiple backcrosses to Col-0)	[1]
sag101	Col-0 *sag101-3* (GABI-Kat 476E10)	[2]
pad4	Col-0 *pad4-1*	[3]
pad4 sag101	*pad4-1 sag101-3*	[2]
eds1 pad4 sag101	*eds1-2 pad4-1 sag101-3*	[4]
sid2	*sid2-1*	[5]
eds1 sid2	*eds1-2 sid2-1*	[2]
pad4 sid2	*pad4-1 sid2-1*	[2]
sag101 sid2	*sag101-3 sid2-1*	This study, cross between *eds1-2 sag101-3 pad4-1* and *sid2-1*
pad4 sag101 sid2	*pad4-1 sag101-3 sid2-1*	This study, cross between *eds1-2 sag101-3 pad4-1* and *sid2-1*
n2	*nrg1.1 nrg1.2*	[6]
a3	*adr1 adr1-L1 adr1-L2*	[7]
n2 a3	*nrg1.1 nrg1.2 adr1 adr1-L1 adr1-L2*	[6]
n2 sid2	*nrg1.1_nrg1.2_sid2-1*	This study, cross between *n2 a3* and *sid2*
a3 sid2	*adr1 adr1-L1 adr1-L2 sid2-1*	
a3 n2 sid2	*nrg1.1 nrg1.2 adr1 adr1-L1 adr1-L2 sid2-1*	
sag101 n2	*nrg1.1 nrg1.2 sag101-3*	This study, cross between *n2 a3* and *eds1 sag101 pad4*
pad4 n2	*nrg1.1 nrg1.2 pad4-1*	
sag101 a3	*adr1 adr1-L1 adr1-L2 sag101-3*	
pad4 a3	*adr1 adr1Ll1 adr1-L2 pad4-1*	
sag101 pad4 a3	*adr1 adr1-L1 adr1-L2 sag101-3 pad4-1*	
sag101 pad4 n2	*nrg1.1 nrg1.2 sag101-3 pad4-1*	
mc1	*mc1-1* (GABI-Kat 096A10)	[8]
mc1 sid2	*mc1 sid2-1*	This study, cross between *mc1* and *pad4 sag101 sid2*
sag101 mc1	*sag101-3 mc*	This study, cross between *mc1* and *pad4 sag101 sid2*
sag101 sid2 mc1	*sag101-3 sid2-1 mc1*	This study, cross between *mc1* and *pad4 sag101 sid2*
pad4 mc1	*pad4-1 mc1*	This study, cross between *mc1* and *pad4 sag101 sid2*
pad4 sid2 mc1	*pad4-1 sid2-1 mc1*	This study, cross between *mc1* and *pad4 sag101 sid2*
pEDS1:EDS1-YFP	pXCG *pEDS1:EDS1-YFP*, Col-0/L*er eds1-2*	[9]
p35S:TRB1-GFP	pAM-PAT p35S:TRB1-GFP Col-0 *trb1-1*	[10]
p35:StrepII-3xHA-YFP	pAM-PAT StrepII-3xHA-YFP Col-0	[6]
pSAG101:SAG101-YFP	pXCG pSAG101:SAG101-YFP, *pad4-1 sag101-3*	This study
pPAD4:PAD4-YFP	pAlligator2 pPAD4:YFP-PAD4, *pad4-1 sag101-3*	This study
pNRG1.2:NRG1.2-HF	pNRG1.2:NRG1.2-6xHis-3xFLAG, Ws-2 *nrg1a nrg1b*	[11]
Nb epss	*N. benthamiana eds1a pad4 sag101a sag101b*	[6]
N. bethamiana wild type	*N. benthamiana* (MPIPZ stock)	[6]

Supplementary Table 2. *Agrobacteria* strains used in this study

Strain name	Full name	Strain
dDL276	p35S:NRG1.1-StrepII-3xHA	GV3101 pMP90RK [6]
dXS323	p35S:NRG1.1$^{E14A/E27A}$-3xHA	GV3101 pMP90RK
dXS318	p35S:NRG1.1$^{L21A/K22A}$-3xHA	GV3101 pMP90RK
dXS320	p35S:NRG1.1^{G199AK200AT201A}-3xHA	GV3101 pMP90RK
dJDQ367	p35S:EDS1-3xFLAG	GV3101 pMP90RK
dXS334	p35S:EDS1^{H476Y}-3xFLAG	GV3101 pMP90RK
dXS338	p35S:EDS1^{F419E}-3xFLAG	GV3101 pMP90RK
MW30	pEDS1:EDS1-YFP	GV3101 pMP90RK
dXS298	p35S:SAG101-3xFLAG	GV3101 pMP90RK
dDL336	pADR1-L2:ADR1_L2- StrepII-3xHA	GV3101 pMP90RK [6]
dXS264	p35S:3xFLAG-GUS	GV3101 pMP90RK
CLR071	p35S:PAD4-3xFLAG	GV3101 pMP90RK

Supplementary Methods

Preparation of peptides for LC-MS/MS analysis

In IP experiments with EDS1-YFP and TRB1-GFP complementation lines, immunoprecipitated proteins in Tris-Urea were reduced with dithiothreitol, alkylated with chloroacetamide, and digested with trypsin (1:100) o/n. Samples were desalted using stage tips with C18 Empore disk membranes (3 M) [12].

IP experiments with YFP-PAD4 and SAG101-YFP complementation lines, proteins (from GFP-trapA enrichment) were submitted to an on-bead digestion. In brief, dry beads were re-dissolved in 25 µL digestion buffer 1 (50 mM Tris, pH 7.5, 2M urea, 1mM DTT, 5 ng/µL trypsin) and incubated for 30 min at 30°C in a Thermomixer with 400 rpm. Next, beads were pelleted, and the supernatant was transferred to a fresh tube. Digestion buffer 2 (50 mM Tris, pH 7.5, 2M urea, 5 mM CAA) was added to the beads, after mixing the beads were pelleted, the supernatant was collected and combined with the previous one. The combined supernatants were then incubated o/n at 32 °C in a Thermomixer with 400 rpm; samples were protected from light during incubation. The digestion was stopped by adding 1 µL TFA and desalted with C18 Empore disk membranes according to the StageTip protocol [12].

Label-free LC-MS/MS data acquisition and data processing

For IP experiments with EDS1-YFP, YFP-PAD4, SAG101-YFP and TRB1-GFP, dried peptides were re-dissolved in 2% ACN, 0.1% TFA (10 µL) for analysis and measured without dilution. Samples were analysed using an EASY-nLC 1200 (Thermo Fisher Scientific) coupled to a Q Exactive Plus mass spectrometer (Thermo Fisher Scientific). Peptides were separated on 16 cm frit-less silica emitters (New Objective, 0.75 µm inner diameter), packed in-house with reversed-phase ReproSil-Pur C18 AQ 1.9 µm resin (Dr. Maisch). Peptides were loaded on the column and eluted for 115 min using a segmented linear gradient of 5% to 95% solvent B (0 min: 5%B; 0-5 min -> 5%B; 5-65 min -> 20%B; 65-90 min ->35%B; 90-100 min -> 55%; 100-105 min ->95%, 105-115 min ->95%) (solvent A 0% ACN, 0.1% FA; solvent B 80% ACN, 0.1%FA) at a flow rate of 300 nL/min. Mass spectra were acquired in data-dependent acquisition mode with a TOP15 method. MS spectra were acquired in the Orbitrap analyser with a mass range of 300–1750 m/z at a resolution of 70,000 FWHM and a target value of 3×106 ions. Precursors were selected with an isolation window of 1.3 m/z. HCD fragmentation was performed at a normalized collision energy of 25. MS/MS spectra were acquired with a target value of 105 ions at a resolution of 17,500 FWHM, a maximum injection time (max.) of 55 ms and a fixed first mass of m/z 100. Peptides with a charge of +1, greater than 6, or with

unassigned charge state were excluded from fragmentation for MS2, dynamic exclusion for 30s prevented repeated selection of precursors.

Raw data were processed using MaxQuant software (version 1.6.3.4, http://www.maxquant.org/) [13] with label-free quantification (LFQ) and iBAQ enabled [13, 14]. MS/MS spectra were searched by the Andromeda search engine against a combined database containing the sequences from A. thaliana (TAIR10_pep_20101214; ftp://ftp.arabidopsis.org/home/tair/Proteins/TAIR10_protein_lists/) and sequences of 248 common contaminant proteins and decoy sequences. Trypsin specificity was required and a maximum of two missed cleavages allowed. Minimal peptide length was set to seven amino acids. Carbamidomethylation of cysteine residues was set as fixed, oxidation of methionine and protein N-terminal acetylation as variable modifications. Peptide-spectrum-matches and proteins were retained if they were below a false discovery rate of 1%. Statistical analysis of the MaxLFQ values was carried out using Perseus (version 1.5.8.5, http://www.maxquant.org/). Quantified proteins were filtered for reverse hits and hits “identified by site” and MaxLFQ values were $\log_2$ transformed. After grouping samples by condition only those proteins were retained for the subsequent analysis that had three valid values in one of the conditions. Two-sample t-tests were performed using a permutation-based FDR of 5%. Alternatively, quantified proteins were grouped by condition and only those hits were retained that had 4 valid values in one of the conditions. The imputed missing values were based on the minimum detected protein abundances in the sample to retain a normally distributed dataset for statistical analyses (1.8 downshift, separately for each column) [15]. Volcano plots were generated in Perseus using an FDR of 5% and an S0=1. The Perseus output was exported and further processed using Excel.

LC-MS/MS analysis of NRG1.2-copurified proteins

Samples were resolved by SDS-PAGE with RunBlueTM 4-20% TEO-Tricine (BCG42012) and stained with InstantBlue® Coomassie Protein Stain (ab119211). Bands were excised from gel with sterile blade and stored at – 20 °C if not submitted fresh. LC-MS and data processing was carried out as previously described [16]. Data was analysed as total spectrum counts in Scaffold Viewer (Proteome Software, v. 4.10) and filtered for a protein threshold probability > 99%, peptide threshold probability > 95%, and a minimum of two peptides identified.

Supplementary References

1. Bartsch M*, et al.* Salicylic acid-independent ENHANCED DISEASE SUSCEPTIBILITY1 signaling in Arabidopsis immunity and cell death is regulated by the monooxygenase FMO1 and the Nudix hydrolase NUDT7. *The Plant cell* **18**, 1038-1051 (2006).

2. Cui H, Gobbato E, Kracher B, Qiu J, Bautor J, Parker JE. A core function of EDS1 with PAD4 is to protect the salicylic acid defense sector in Arabidopsis immunity. *The New phytologist* **213**, 1802-1817 (2017).

3. Jirage D*, et al.* Arabidopsis thaliana PAD4 encodes a lipase-like gene that is important for salicylic acid signaling. *Proceedings of the National Academy of Sciences of the United States of America* **96**, 13583-13588 (1999).

4. Wagner S*, et al.* Structural basis for signaling by exclusive EDS1 heteromeric complexes with SAG101 or PAD4 in plant innate immunity. *Cell host & microbe* **14**, 619-630 (2013).

5. Wildermuth MC, Dewdney J, Wu G, Ausubel FM. Isochorismate synthase is required to synthesize salicylic acid for plant defence. *Nature* **414**, 562-565 (2001).

6. Lapin D*, et al.* A Coevolved EDS1-SAG101-NRG1 Module Mediates Cell Death Signaling by TIR-Domain Immune Receptors. *The Plant cell* **31**, 2430-2455 (2019).

7. Bonardi V, Tang S, Stallmann A, Roberts M, Cherkis K, Dangl JL. Expanded functions for a family of plant intracellular immune receptors beyond specific recognition of pathogen effectors. *Proceedings of the National Academy of Sciences of the United States of America* **108**, 16463-16468 (2011).

8. Coll NS, Smidler A, Puigvert M, Popa C, Valls M, Dangl JL. The plant metacaspase AtMC1 in pathogen-triggered programmed cell death and aging: functional linkage with autophagy. *Cell Death & Differentiation* **21**, 1399-1408 (2014).

9. García AV*, et al.* Balanced nuclear and cytoplasmic activities of EDS1 are required for a complete plant innate immune response. *PLoS pathogens* **6**, e1000970 (2010).

10. Zhou Y, Hartwig B, James GV, Schneeberger K, Turck F. Complementary Activities of TELOMERE REPEAT BINDING Proteins and Polycomb Group Complexes in Transcriptional Regulation of Target Genes. *The Plant cell* **28**, 87-101 (2016).

11. Castel B*, et al.* Diverse NLR immune receptors activate defence via the RPW8-NLR NRG1. *The New phytologist* **222**, 966-980 (2019).

12. Rappsilber J, Ishihama Y, Mann M. Stop and go extraction tips for matrix-assisted laser desorption/ionization, nanoelectrospray, and LC/MS sample pretreatment in proteomics. *Analytical chemistry* **75**, 663-670 (2003).

13. Cox J, Mann M. MaxQuant enables high peptide identification rates, individualized p.p.b.-range mass accuracies and proteome-wide protein quantification. *Nature biotechnology* **26**, 1367-1372 (2008).

14. Tyanova S, Temu T, Cox J. The MaxQuant computational platform for mass spectrometry-based shotgun proteomics. *Nature protocols* **11**, 2301-2319 (2016).

15. Tyanova S*, et al.* The Perseus computational platform for comprehensive analysis of (prote)omics data. *Nature Methods* **13**, 731-740 (2016).

16. Bender KW*, et al.* Autophosphorylation-based Calcium (Ca(2+)) Sensitivity Priming and Ca(2+)/Calmodulin Inhibition of Arabidopsis thaliana Ca(2+)-dependent Protein Kinase 28 (CPK28). *The Journal of biological chemistry* **292**, 3988-4002 (2017).
