# Supplementary material for: Pathogen effector recognition-dependent association of NRG1 with EDS1 and SAG101 in TNL receptor immunity
Source: Nat Commun. 2021 Jun 7;12:3335. doi: 10.1038/s41467-021-23614-x (PMC8185089; doi:10.1038/s41467-021-23614-x)
Supplement: Supplementary file 3 — Description of Additional Supplementary Files [file 41467_2021_23614_MOESM3_ESM.pdf]

Description of Additional Supplementary Files

File Name: Supplementary Data 1
Description: Oligonucleotide sequences used in this study

File Name: Supplementary Data 2
Description: Normalized estimated abundances of proteins copurified with YFP-PAD4, SAG101-YFP or YFP-SH and their statistical analysis.

Legend: orange and blue – protein group shows significantly increased or reduced enrichment, respectively, in the test group compared to the control group.

File Name: Supplementary Data 3
Description: Normalized estimated abundances of proteins copurified with EDS1-YFP or TRB1-GFP and their statistical analysis

Legend: yellow – protein group shows significant enrichment in the test group compared to the control groups; black – putative contaminants

File Name: Supplementary Data 4
Description: Peptide counts for proteins copurified with NRG1.2-HF

Legend: Pf0-1 avrRps4 – effector tester strain *Pseudomonas fluorescens* 0-1 EtHAn delivering avrRps4 effector; Pf0-1 EV – effector tester strain *Pseudomonas fluorescens* 0-1 EtHAn with the empty vector; rep1 and rep2 – replicate 1 and 2, respectively.
