# Supplementary material for: Pathogen effector recognition-dependent association of NRG1 with EDS1 and SAG101 in TNL receptor immunity
Source: Nat Commun. 2021 Jun 7;12:3335. doi: 10.1038/s41467-021-23614-x (PMC8185089; doi:10.1038/s41467-021-23614-x)
Supplement: Supplementary file 8 — Reporting Summary [file 41467_2021_23614_MOESM8_ESM.pdf]

nature research

Corresponding author(s): Jane E. Parker, Jonathan D.G. Jones

Last updated by author(s): 2021/04/13

Reporting Summary

Nature Research wishes to improve the reproducibility of the work that we publish. This form provides structure for consistency and transparency in reporting. For further information on Nature Research policies, see our Editorial Policies and the Editorial Policy Checklist.

Statistics

For all statistical analyses, confirm that the following items are present in the figure legend, table legend, main text, or Methods section.

n/a	Confirmed	
☐	☒	The exact sample size (*n*) for each experimental group/condition, given as a discrete number and unit of measurement
☐	☒	A statement on whether measurements were taken from distinct samples or whether the same sample was measured repeatedly
☐	☒	The statistical test(s) used AND whether they are one- or two-sided<br>*Only common tests should be described solely by name; describe more complex techniques in the Methods section.*
☐	☒	A description of all covariates tested
☐	☒	A description of any assumptions or corrections, such as tests of normality and adjustment for multiple comparisons
☐	☒	A full description of the statistical parameters including central tendency (e.g. means) or other basic estimates (e.g. regression coefficient) AND variation (e.g. standard deviation) or associated estimates of uncertainty (e.g. confidence intervals)
☐	☒	For null hypothesis testing, the test statistic (e.g. *F*, *t*, *r*) with confidence intervals, effect sizes, degrees of freedom and *P* value noted<br>*Give P values as exact values whenever suitable.*
☒	☐	For Bayesian analysis, information on the choice of priors and Markov chain Monte Carlo settings
☒	☐	For hierarchical and complex designs, identification of the appropriate level for tests and full reporting of outcomes
☒	☐	Estimates of effect sizes (e.g. Cohen's *d*, Pearson's *r*), indicating how they were calculated

Our web collection on statistics for biologists contains articles on many of the points above.

Software and code

Policy information about availability of computer code

Data collection	Perseus (version 1.5.8.5), MaxQuant software (version 1.6.3.4), Scaffold (Proteome Software, version 4.10)
Data analysis	Statistical analysis was performed in R (3.6.1 through 4.0.2) using base ANOVA and posthoc tests. Heatmaps were generated with the pheatmap (1.0.12) package in R. Boxplots and barplots were generated with the ggplot2 (3.3.2) package in R. Multiple sequence alignment in Supplemental Figure 7 was prepared with msa package (1.22.0) in R. NRG1.1 structure model was prepared with SWISS-MODEL (ref. 69; SMTL version 2019-05-22, PDB release 2019-05-17; ProMod3 v. 1.3.0). Visualization of the structures was performed in Pymol (2.3.4 - 2.4.1). Excel in Microsoft Office suites 2016, 2019, 365 were used for the analysis of processed mass spectrometry data. Custom scripts for statistical analysis described in GitHub repository for Lapin et al 2019 (ref. 26 in the main text) are also available at Zenodo https://zenodo.org/record/4660032#.YGcSAy0Rq_w

For manuscripts utilizing custom algorithms or software that are central to the research but not yet described in published literature, software must be made available to editors and reviewers. We strongly encourage code deposition in a community repository (e.g. GitHub). See the Nature Research guidelines for submitting code & software for further information.

Data

Policy information about availability of data

All manuscripts must include a data availability statement. This statement should provide the following information, where applicable:

- Accession codes, unique identifiers, or web links for publicly available datasets
- A list of figures that have associated raw data
- A description of any restrictions on data availability

Mass spectrometry data are available in the PRoteomics IDEntification database under accession numbers PXD025202 (PRIDE, [https://www.ebi.ac.uk/pride/archive/projects/PXD025202]), PXD021301 (PRIDE, [https://www.ebi.ac.uk/pride/archive/projects/PXD021301]), PXD022907 (PRIDE, [https://www.ebi.ac.uk/pride/

archive/projects/PXD022907]). Source data are provided in this paper. All relevant data are also available from the authors.
Accession numbers of NLRs in the alignment in Figure S7: NRG1.1 – AT5G66900.1 (TAIR, [https://www.arabidopsis.org/servlets/TairObject?type=locus&name=At5g66900]), NRG1.2 – AT5G66910.1 (TAIR, [https://www.arabidopsis.org/servlets/TairObject?type=locus&name=At5g66910]), LusNRG1 - Lus10022464 (Phytozome, [https://phytozome.jgi.doe.gov/pz/portal.html#!gene?search=1&detail=1&method=3127&searchText=transcriptid:23171720]), NbNRG1 - Niben101Scf02118g00018.1 (SolGenomics, [https://solgenomics.net/jbrowse_solgenomics/?data=data%2Fjson%2FNiben1.0.1&loc=Niben101Scf02118%3A107051..119466&tracks=DNA%2CNibenv101_gene_models&highlight=]), AtADR1-L2 - AT5G04720.1 (TAIR, [https://www.arabidopsis.org/servlets/TairObject?type=locus&name= At5g04720]), SlADR1 - Solyc04g079420.3.1 (SolGenomics, [https://solgenomics.net/locus/110992/view]), NbADR1 - Niben101Scf02422g02015.1 (SolGenomics, [https://solgenomics.net/jbrowse_solgenomics/?data=data%2Fjson%2FNiben1.0.1&loc=Niben101Scf02422%3A298959..305123&tracks=DNA%2CNibenv101_gene_models&highlight=]), SlNRC4 - Solyc04g007070.3.1 (SolGenomics, [https://solgenomics.net/locus/20361/view]), SlNRC3 - XP_004238948.1 (NCBI, [https://www.ncbi.nlm.nih.gov/protein/XP_004238948.1/]), AtZAR1 - AT3G50950.2 (TAIR, [https://www.arabidopsis.org/servlets/TairObject?type=locus&name= AT3G50950]), N - Q40392 (Uniprot, [https://www.uniprot.org/uniprot/Q40392]), Roq1 - ATD14363.1 (NCBI, [https://www.ncbi.nlm.nih.gov/protein/ATD14363.1]), RPP4 - F4JNA9 (Uniprot, [https://www.uniprot.org/uniprot/F4JNA9]), RPS4 - Q9XGM3 (Uniprot, [https://www.uniprot.org/uniprot/Q9XGM3]), RPM1 - Q39214 (Uniprot, [https://www.uniprot.org/uniprot/Q39214]), Rx - Q9XGF5 (Uniprot, [https://www.uniprot.org/uniprot/ Q9XGF5]).
The CryoEM structure of AtZAR1 used as a template – 6j5t (PDB, [https://www.rcsb.org/structure/6J5T]). EDS1-SAG101 X-ray structure – 4nfu (PDB, [https://www.rcsb.org/structure/4NFU])

Links to databases:
PRIDE www.ebi.ac.uk/pride/
PDB: www.rcsb.org
TAIR: www.arabidopsis.org
Phytozome: www.phytozome.jgi.doe.gov
Uniprot: www.uniprot.org
SolGenomics: www.solgenomics.net

Code availability
Custom scripts for statistical analysis described in GitHub repository for Lapin et al 2019 (ref. 26 in the main text) are also available at Zenodo https://zenodo.org/record/4660032#.YGcSAy0Rq_w

Field-specific reporting

Please select the one below that is the best fit for your research. If you are not sure, read the appropriate sections before making your selection.

☒ Life sciences ☐ Behavioural & social sciences ☐ Ecological, evolutionary & environmental sciences

For a reference copy of the document with all sections, see nature.com/documents/nr-reporting-summary-flat.pdf

Life sciences study design

All studies must disclose on these points even when the disclosure is negative.

Sample size	no mathematical methods were used to predetermine the sample size; sample sizes were based on previous experience described in Lapin et al. 2019 (ref. 26 in the main text) and Castel et al. 2019 (ref. 19 in the main text).
Data exclusions	no collected data points were excluded from the analysis
Replication	For the analysis of quantitative data, measurements from individual samples (replicates) in independent experiments were pooled before the statistical analysis, and experiment was then included as a factor into the ANOVA model. The number of times experiments in question were repeated is mentioned in the figure legends. For qualitative (Western blotting) analysis, the number of experiments in which a similar result was observed is stated in figure legends.
Randomization	Randomization was not performed explicitly. Plants of different genotypes were distributed over several trays, and trays were placed in different locations in the growth chamber in different experiments to avoid the position effect.
Blinding	Investigators were not blunded during the data collection because otherwise efficient processing of the large number of individual samples would not be feasible. For MS experiments, blinding is not feasible since information about the sample identity must be provided before the sample submission for processing in Proteomics facilities.

Reporting for specific materials, systems and methods

We require information from authors about some types of materials, experimental systems and methods used in many studies. Here, indicate whether each material, system or method listed is relevant to your study. If you are not sure if a list item applies to your research, read the appropriate section before selecting a response.

Materials & experimental systems

n/a	Involved in the study
[ ]	[x] Antibodies
[x]	[ ] Eukaryotic cell lines
[x]	[ ] Palaeontology and archaeology
[x]	[ ] Animals and other organisms
[x]	[ ] Human research participants
[x]	[ ] Clinical data
[x]	[ ] Dual use research of concern

Methods

n/a	Involved in the study
[x]	[ ] ChIP-seq
[x]	[ ] Flow cytometry
[x]	[ ] MRI-based neuroimaging

Antibodies

Antibodies used

anti-GFP (Millipore, Sigma,11814460001; clones 7.1 and 13.1), anti-HA (Cell Signalling, #3724, clone c29f4), anti-FLAG (Millipore, Sigma, F1804, clone M2), anti-EDS1 (Agrisera, AS13 2751, polyclonal), anti-FLAG (Sigma, A8592, clone M2), secondary HRP-conjugated antibodies: A0545, A9044 and A6154 (Millipore, Sigma), sc-2006 and sc-2005 (Santa Cruz). Lot numbers were recorded for the used antibodies.

Validation

The specificity of primary antibodies was established in Arabidopsis thaliana and Nicotiana benthamiana by including respective negative controls in the Western blot in this publication, Lapin et al., 2019 (The Plant Cell; doi.org/10.1105/tpc.19.00118, ref. 26 in the main text), and Castel et al., 2019 (New Phytologist; doi.org/10.1111/nph.15659, ref. 19 in the main text)

Further evidence of validation:

anti-GFP (Millipore, Sigma,11814460001, clones 7.1 and 13.1) - bulletin version 07, section "Western Blot". "When incubated with the blot membrane at a concentration of 0.4 ug/ ml, the Anti-GFP antibody binds specifically to the recombinant GFP fusion protein. In contrast, a negative control E. coli extract (without GFP fusion protein) shows no significant nonspecific binding of conjugate under standard conditions."

anti-HA (Cell Signalling, #3724, clone c29f4) - product documentation, validated in Western blot analysis of extracts from HeLa cells, untransfected or transfected with either HA-FoxO4 or HA-Akt3, expected to work in all species (https://www.cellsignal.com/products/primary-antibodies/ha-tag-c29f4-rabbit-mab/3724)

anti-FLAG (Millipore, Sigma, F1804, clone M2) - bulletin, section "Reagent/Specificity" - The monoclonal antibody detects only the target protein band(s) on a Western blot from an E. coli, plant or mammalian crude cell lysate.

anti-EDS1 (Agrisera, AS13 2751, polyclonal) - product information from supplier, validated in Western blot with the eds1-2 mutant as a negative control (courtesy of Morgan K. Halane, University of Missouri, USA; https://www.agrisera.com/en/artiklar/eds1-enhanced-disease-susceptibility-1.html); reacts with Arabidopsis thaliana but not Nicotiana benthamiana EDS1.

anti-FLAG (Sigma, A8592, clone M2) - see note for anti-FLAG (Millipore, Sigma, F1804, clone M2).
